# New Oral Anticoagulants Are Not Superior to Warfarin in Secondary Prevention of Stroke or Transient Ischemic Attacks, but Lower the Risk of Intracranial Bleeding: Insights from a Meta-Analysis and Indirect Treatment Comparisons 

**DOI:** 10.1371/journal.pone.0077694

**Published:** 2013-10-25

**Authors:** Partha Sardar, Saurav Chatterjee, Wen-Chih Wu, Edgar Lichstein, Joydeep Ghosh, Shamik Aikat, Debabrata Mukherjee

**Affiliations:** 1 Department of Medicine, New York Medical College, Metropolitan Hospital Center, New York, New York, United States of America; 2 Brown University and the Providence VAMC, Providence, Rhode Island, United States of America; 3 Maimonides Medical Center, Brooklyn, New York, United States of America; 4 Division of Cardiology, Gill Heart Institute, University of Kentucky, Lexington, Kentucky, United States of America; 5 Texas Tech University Health Sciences Center, El Paso, Texas, United States of America; Charité Universitaetsmedizin Berlin, Germany

## Abstract

**Purpose:**

Patients with Atrial Fibrillation (AF) and prior stroke are classified as high risk in all risk stratification schemes. A systematic review and meta-analysis was performed to compare the efficacy and safety of New Oral Anticoagulants (NOACs) to warfarin in patients with AF and previous stroke or transient ischemic attack (TIA).

**Methods:**

Three randomized controlled trials (RCTs), including total 14527 patients, comparing NOACs (apixaban, dabigatran and rivaroxaban) with warfarin were included in the analysis. Primary efficacy endpoint was ischemic stroke, and primary safety endpoint was intracranial bleeding. Random-effects models were used to pool efficacy and safety data across RCTs. RevMan and Stata software were used for direct and indirect comparisons, respectively.

**Results:**

In patients with AF and previous stroke or TIA, effects of NOACs were not statistically different from that of warfarin, in reduction of stroke (Odds Ratio [OR] 0.86, 95% confidence interval [CI] 0.73- 1.01), disabling and fatal stroke (OR 0.85, 95% CI 0.71-1.04), and all-cause mortality (OR 0.90, 95% CI 0.79 -1.02). Randomization to NOACs was associated with a significantly lower risk of intracranial bleeding (OR 0.42, 95% CI 0.25-0.70). There were no major differences in efficacy between apixaban, dabigatran (110 mg BID and 150 mg BID) and rivaroxaban. Major bleeding was significantly lower with apixaban and dabigatran (110 mg BID) compared with dabigatran (150 mg BID) and rivaroxaban.

**Conclusion:**

NOACs may not be more effective than warfarin in the secondary prevention of ischemic stroke in patients with a prior history of cerebrovascular ischemia, but have a lower risk of intracranial bleeding.

## Introduction

Patients with atrial fibrillation (AF) who have had a history of previous stroke or transient ischemic attack (TIA) are at an increased risk of recurrent stroke and or systemic embolism. AF patients with prior cerebral ischemia are classified as high risk by almost all stroke risk stratification schemes [1-3]. Following the initial attack of stroke, the recurrence rate of stroke varies between 2% and 15% in the first year, and 5% yearly thereafter [4]. In patients with moderate to high-risk AF, therapeutic anticoagulation has been shown to reduce the risk of embolic phenomena and mortality [5]. Previous studies have shown the beneficial effect of warfarin in the prevention of stroke in AF, including patients with previous stroke or TIA [6]. However use of warfarin is associated with several limitations including a narrow therapeutic range, need for coagulation monitoring, drug - food interactions, and an increased risk of hemorrhage, including brain hemorrhage [6]. Newer drugs, direct thrombin inhibitor dabigatran and factor Xa inhibitors rivaroxaban and apixaban have been developed as an alternative to warfarin. Recent trials have compared the efficacy of these newer oral anticoagulants (NOACs) with warfarin in patients with AF and previous history of stroke or TIA [7-9]. 

A subgroup analysis of the RE-LY (Randomized Evaluation of Long-Term Anticoagulation Therapy) trial has compared the effects of dabigatran with warfarin in patients with atrial fibrillation and previous stroke or TIA [7]. The study concluded that in comparison to warfarin, 110 mg dabigatran was non-inferior and 150 mg dabigatran was better in prevention of stroke or systemic embolism. Another significant finding was that 110 mg dabigatran caused less major bleeding and 150 mg dabigatran had similar rates of major bleeding compared to warfarin. Subgroup analysis of ROCKET AF trial (Rivaroxaban Once Daily Oral Direct Factor Xa Inhibitor Compared with Vitamin K Antagonism for Prevention of Stroke and Embolism Trial in Atrial Fibrillation) showed similar beneficial effect of rivaroxaban in patients with or without previous stroke or TIA [8]. Rivaroxaban (20 mg once daily) was non-inferior to warfarin in the prevention of stroke and systemic embolism in AF patients. The subgroup analysis of the ARISTOTLE (Apixaban for Reduction in Stroke and Other Thromboembolic Events in Atrial Fibrillation) analysis revealed that apixaban 5 mg twice daily was superior to warfarin for the prevention of stroke or systemic embolism in AF patients with previous stroke [9]. Apixaban was also associated with significantly less major bleeding. 

Although these trials suggested in favor of NOACs in secondary prevention of stroke in patients with AF and previous stroke and TIA, the reported efficacy outcomes and safety outcomes were heterogeneous and also inconclusive on few occasions. At the same time there is no previous or ongoing, head-to-head trial among these NOACs. So the data on relative efficacy and safety of NOACs, compared against each other are very limited. 

We systematically reviewed the data from randomized controlled trials of the new oral anticoagulants in patients with AF and previous stroke or transient ischemic attack. We performed pooled direct comparisons with warfarin and indirect comparisons among the NOACs on the efficacy and safety outcomes data. 

## Methods

We systematically searched the published literature for trials comparing any of the new oral anticoagulants (dabigatran, rivaroxaban, and apixaban) with warfarin in patients with AF and previous stroke and TIA.

### Data Sources and Searches

The authors searched the PubMed, Cochrane CENTRAL, EMBASE and CINAHL databases for English language, peer-reviewed publications comparing NOACs with warfarin from January 2001 (NOACs were introduced that year) through September 2012. We used the following Medical Subject Heading terms and/or keywords: “new oral anticoagulants,” “oral thrombin inhibitors,” “oral factor Xa inhibitors,” “dabigatran,” “rivaroxaban,” “apixaban”. Clinical trial databases, relevant reviews, and the reference lists of all retrieved reports were manually searched for potentially relevant studies not identified in our initial electronic database search. 

### Study Selection

The PRISMA (Preferred Reporting Items for Systematic reviews and Meta-Analyses) statement for reporting systematic reviews and meta-analyses of RCTs [10] was used as a reference method for this study. The studies were included if they were RCTs, included atrial fibrillation patients with and without previous history of stroke or TIA, randomized subjects to warfarin and simultaneously to rivaroxaban, dabigatran, or apixaban. We included both open-label and blinded studies, as dose monitoring of warfarin makes blinding very difficult. To assess the long-term efficacy and safety of NOACs, only RCTs with long term follow-up (at least one year) were included in our analysis. 

### Data Extraction and Quality Assessment

Two authors (PS, SC) reviewed the trials, ensured that they met the inclusion criteria and abstracted the data. This was checked for accuracy by the other authors and disagreements were resolved by consensus (8% of the time). We performed objective assessment of the trials using the method specified in the Cochrane Handbook of Systematic Reviews—assessing for randomization, allocation concealment, comparability of groups at baseline, blinding, concomitant interventions, completeness of follow-up and differential loss to follow-up, whether incomplete data were addressed appropriately, validity of outcome measures, and conflicts of interest. 

### Data Synthesis and Analysis

#### Outcome measures

The main efficacy outcome of interest was an ischemic stroke. Other efficacy outcomes were stroke or systemic embolism, hemorrhagic stroke, disabling and fatal stroke and all-cause mortality. The main safety outcome of interest was intracranial bleeding. Other safety outcomes were major bleeding, and gastrointestinal bleeding. 

#### Statistical analysis

We performed direct pooled comparisons between dabigatran, rivaroxaban, and apixaban versus warfarin as well as indirect comparisons between the three drugs (with warfarin as common comparator) on an intention to treat basis. Odds Ratio (OR) and their respective 95% confidence intervals (CI) were estimated for each study and for the analysis for each of the oral anticoagulants. We assessed the heterogeneity using the Cochran Q test [11] and the Higgins I^2^ test [12]. A Cochran’s Q P<0.10 and I^2^ >50% were considered to show significant heterogeneity in this meta-analysis [12]. Random effects model described by Der-Simonian and Laird was used for our main analysis [13]. We also confirmed our results with use of a fixed effects model described by Mantel Haenszel, in the absence of heterogeneity [14]. We created funnel plots showing the standard error and the effect size to evaluate publication bias, and also assessed the same quantitatively with the regression test of Egger. Direct comparisons were performed using the Review Manager Version 5.1 (The Nordic Cochrane Center, The Cochrane Collaboration, 2008, Copenhagen). 

As a preferred method for indirect comparison, we used Bucher’s method [15] for comparisons across the trials. Bucher’s method estimates the comparative effectiveness of two or more treatments by analyzing the extent of relative treatment effects against a common comparator. The application of this indirect comparison method is based on a similarity assumption. Bucher’s method relies on the assumption that the trials or subgroups within trials are comparable in respect to clinical moderators (patient characteristics, interventions, follow up periods) and methodological moderators (randomization and blinding). We used this approach to evaluate the relative efficacy and safety of apixaban, dabigatran (110 mg & 150 mg), and rivaroxaban using warfarin as common comparator. 

To assess whether differences in trial methodology and protocol, as well as patient characteristics affected the outcomes, random-effects meta-regression analyses were conducted. For indirect comparisons [Bucher’s method], and meta-regression analyses we used the Stata 11.2SE (StataCorp LP, College Station, Texas) software. 

## Results

**Figure 1:**
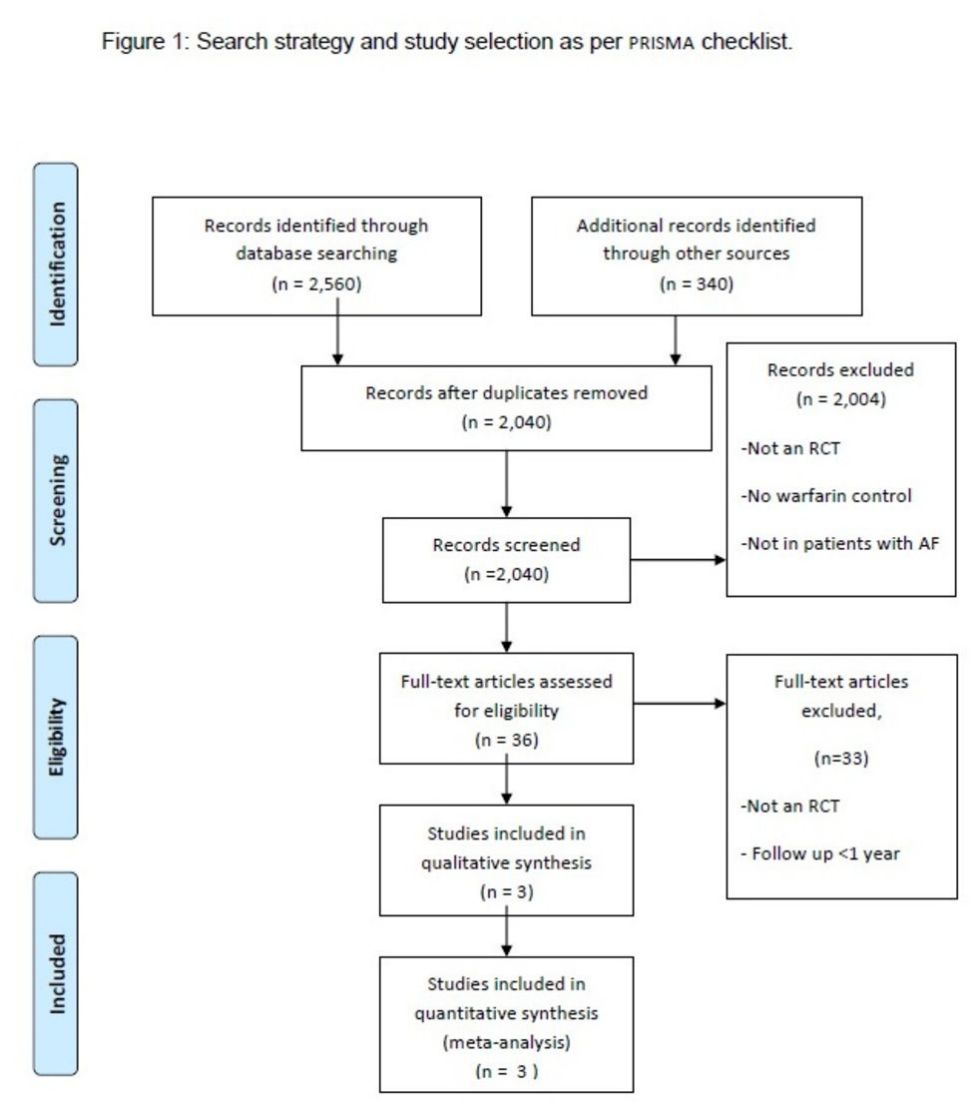
Search strategy and study selection as per PRISMA checklist.

A total of 2,560 reports were identified by our electronic database search (Figure 1). Another 340 reports were also identified through other sources. After removing duplicates, non-relevant publications, and other reasons for exclusion as mentioned in Figure 1, we accessed 36 full text articles for eligibility. Finally, three trials involving total 14527 patients with previous stroke or TIA met our inclusion criteria and were included in the present analysis. All the trials were initially reported with AF patients including all the risk groups [16-18]. Subsequently the data including the detailed sub-group analysis of AF patients with previous stroke or TIA were published [7-9]. For our analysis we included data from three sub-group analyses of all three initial reports. The three included trials assessed the relative efficacy and safety of new oral anticoagulants, dabigatran, apixaban, or rivaroxaban compared to warfarin in patients with AF and previous stroke or TIA (Table 1). 

**Table 1:** Study and patient characteristics (AF patients with previous stroke or TIA) of included randomized trials.

	**Trial**		
**Study Characteristics**	RE-LY	ROCKET AF	ARISTOTLE
**Total (n) of Previous stroke or TIA (%of total population)**	3623(20%)	7468 (52%)	3436 (19%)
**Total population in the study**	18113	14264	18201
**Mean TTR,%**	63	57.1	65
**Follow up periods**	2.0 years[IQR 1.14 -2.86]	Median duration, 676 days (510-845)	1.8 years [IQR-1.4-2.3]
**Study Design**	Prospective Randomized, open label, blinded endpoint (PROBE)	Multicenter, randomized, double-blind, double-dummy	Randomized control, double-blind, parallel arm
**Stroke related exclusion Criteria**	Stroke within 14 days or severe, disabling stroke within 6 months	TIA within 3 days, acute stroke within 14 days, or severe disabling stroke (modified Rankin score 4–5, inclusive) within 3 months of randomization	Patients with a previous intracranial hemorrhage (ICH) or any stroke within 7 days before random assignment
**No of only previous TIA in stroke or TIA group**	1663	2561	956

TIA: Transient Ischemic Attack

These three trials randomized a total of 50578 atrial fibrillation patients, including 14527 patients with previous stroke or TIA. Of the patients with previous stroke and TIA, 5180 had TIA only. The average age of patients with previous stroke and TIA ranged from 70.1 to 71 years, men constituted 61% to 64.1% of the study populations. In RE-LY and ARISTOTLE trials >90% patients with previous stroke and TIA had CHADS_2_≥3, and in ROCKET-AF trial median CHADS_2_ score was 4. The mean time in the therapeutic (TTR) range of warfarin ranged from 57.1% to 65%. The prevalence of prior diabetes and hypertension was highest in ROCKET-AF compared with the other 2 trials. Prior warfarin use among these high risk population was >60% in all three trials- RE-LY, ROCKET-AF and ARISTOTLE. ROCKET-AF and RE-LY excluded patients with acute stroke within 14 days before randomization, and ARISTOTLE excluded any stroke within 7 days before random assignment (Table 1 and Table 2). In each trial, the new oral anticoagulants were found to be superior or non-inferior to warfarin for the composite end point of stroke and systemic embolism. The risk of hemorrhagic stroke was lower with NOACs compared to warfarin. The definitions of efficacy and safety outcomes are mentioned in Table S1.

**Table 2:** Demographics and baseline clinical characteristics (Patients with previous stroke or TIA) of included randomized controlled trials.

Characteristics	Trial					
	RE-LY			ROCKET AF		ARISTOTLE
	Dabigatran 110	Dabigatran 150	Warfarin	Rivaroxaban	Warfarin	
Total (n)	1195	1233	1195	3754	3714	3436
Age(years)	70.2(9.4)	70.8(10.1)	70.4(9.5)	71(64-76)	71(64-77)	70.1(9.5)
Men, %	64.1	62.2	62.4	61	61	63
CHADS_2_≥3, %	90.0	90.2	88.6			92
CHADS_2_ 2, %	10.0	9.8	11.4	Median CHADS_2_ 4(3-5)	Median CHADS_2_ 4(3-5)	8
CHADS_2_ 1, %	0	0	0			0
Vitamin K antagonist naïve, %	43.9	44	45.8	41	41	39
Diabetes,%	22.4	23.7	21.4	25	24	26
Hypertension,%	77	77.3	76.2	85	85	83

Table constructed as per available data in the included trials. For RE-LY and ARISTOTLE trial, Data are mean (SD) or number (%), if not specified. For ROCKET AF trial, Data are median (IQR) or number (%), if not specified. TIA: Transient ischemic attack.

### Direct Comparisons

#### Efficacy outcomes

**Figure 2:**
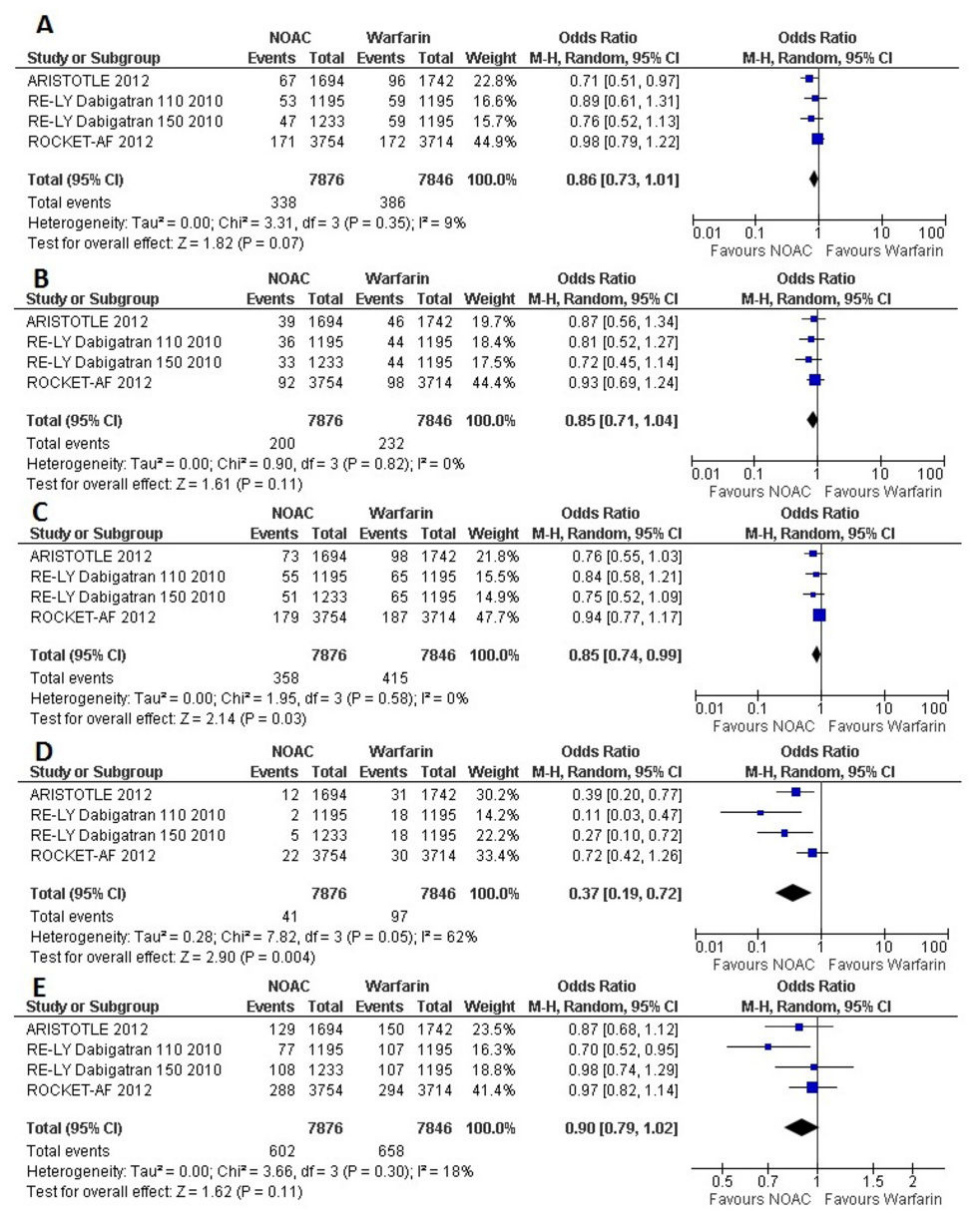
Forest plot(s) comparing NOACs and warfarin in AF patients with previous stroke or TIA, for stroke (A), disabling and fatal stroke(B), stroke and systemic embolism (C), hemorrhagic stroke (D), and all-cause mortality (E).

Efficacy analysis of NOACs compared to warfarin in Atrial Fibrillation patients with previous history of stroke or TIA, showed varied results (Figure 2). When data were pooled across RCTs, No statistically significant difference was found between NOACs and warfarin in the prevention of primary outcome and two other important outcomes, for stroke (Odds Ratio [OR] 0.86, 95% confidence interval [CI] 0.73- 1.01), for disabling and fatal stroke (OR 0.85, 95% CI 0.71-1.04), and for all-cause mortality (OR 0.90, 95% CI 0.79 -1.02). In comparison to warfarin, NOACs only marginally decreased stroke and systemic embolism (OR 0.85, 95% CI 0.74 -0.99). Compared to warfarin most significant beneficial effect of NOACs was found in the prevention of hemorrhagic stroke (OR 0.37, 95% CI 0.19-0.72). 

#### Safety outcomes

**Figure 3:**
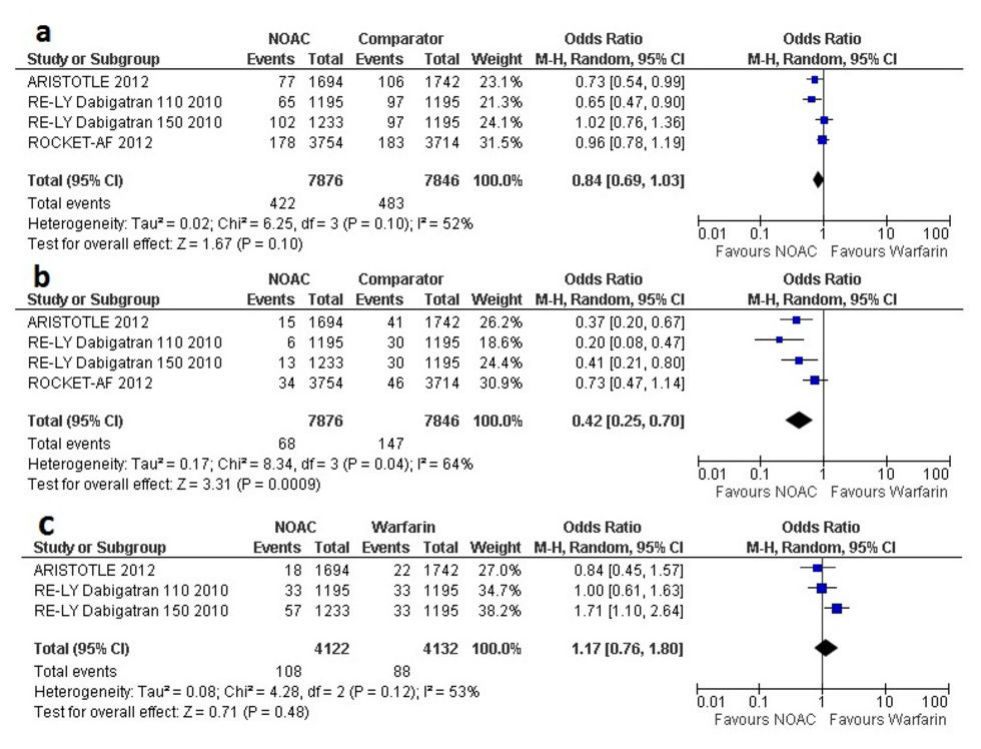
Forest plot(s) comparing NOACs and warfarin in AF patients with previous stroke or TIA, for major bleeding (A), intracranial bleeding (B), and gastrointestinal bleeding(C).

Randomization to NOACs were associated with significantly lower risk for intracranial bleeding (OR 0.42, 95% CI 0.25-0.70) (Figure 3). Major bleeding with NOACs was similar as with warfarin (for AF with stroke and TIA, OR 0.84, 95% CI 0.69 to 1.03). Use of NOACs was related to a statistically non-significant trend towards a higher rate of gastrointestinal major bleeding compared to that of warfarin (OR 1.17, 95% CI 0.76 to 1.80); these results may be considered as inconclusive due to wide confidence intervals. 

**Figure 4:**
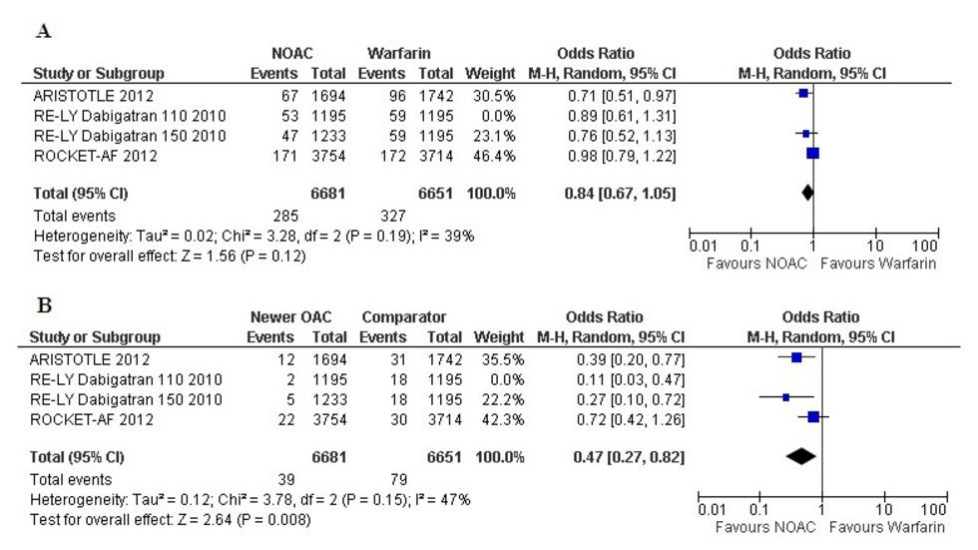
Forest plot comparing NOACs (including only 150 mg dabigatran) and warfarin for stroke (A) and intracranial bleeding (B).

**Figure 5:**
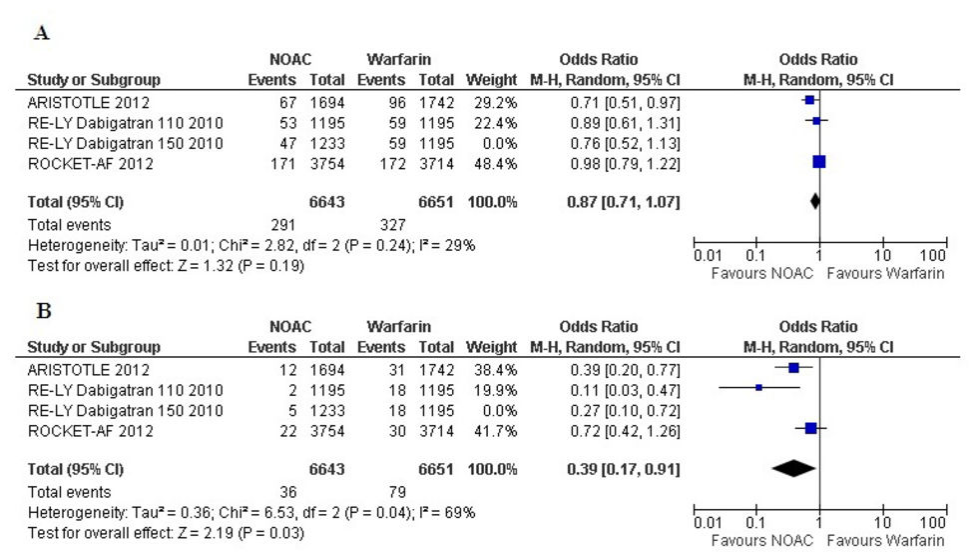
Forest plot comparing NOACs (including only 110 mg dabigatran) and warfarin for stroke (A) and intracranial bleeding (B).

To account for the comparison of the 3-arm RE-LY trial [7] for the 2 doses of dabigatran versus warfarin, we performed separate analyses with each dose as a comparator of warfarin in separate pooled analyses of the primary outcome (Figures 4 and 5). The separate analyses did not show substantial differences with the original pooled outcomes (Figures 2 and 3). 

### Indirect Comparisons

#### Relative efficacy of dabigatran, apixaban, and rivaroxaban

There were no significant differences in efficacy for apixaban versus dabigatran (110 mg BID and 150 mg BID) or rivaroxaban; or rivaroxaban versus dabigatran (both doses) in preventing stroke or systemic embolism (Table 3). Hemorrhagic stroke was significantly less with dabigatran 110 mg BID in comparison to rivaroxaban (OR 0.15; 95% CI 0.03-0.67, p <0.0001), but not with 150 mg BID. For the outcomes of stroke and disabling and fatal stroke, there were no significant differences between the NOACs. 

**Table 3:** Indirect comparisons between apixaban, dabigatran and rivaroxaban with warfarin as single common comparator*.

**Odd Ratio** **(95% CI**)					
**Efficacy End Points**	Apixaban Vs Dabigatran 110	Apixaban Vs Dabigatran 150	Apixaban Vs Rivaroxaban	Dabigatran 110 Vs Rivaroxaban	Dabigatran 150 Vs Rivaroxaban
**Stroke or Systemic Embolism**	0.9(0.56-1.47)	1.01(0.62-1.65)	0.81(0.55-1.18)	0.89(0.59-1.36)	0.8(0.52-1.22)
**Hemorrhagic stroke**	3.55(0.77-16.40)	1.44(0.44-4.77)	0.54(0.23-1.29)	**0.15(0.03-0.67)**	0.37(0.12-1.16)
**Stroke**	0.8(0.48-1.31)	0.93(0.56-1.55)	0.75(0.51-1.11)	0.95(0.61-1.46)	0.81(0.52-1.26)
**Disabling and fatal stroke**	1.07(0.58-2.00)	1.2(0.64-2.29)	0.93(0.55-1.58)	0.87(0.51-1.49)	0.77(0.45-1.34)
**Safety end points**					
**Major Bleeding**	1.12(0.72-1.75)	**0.19(0.13-0.28)**	**0.19(0.14-0.28)**	**0.68(0.46-0.99)**	1.06(0.74-1.52)
**Intracranial Hemorrhage**	1.85(0.63-5.40)	0.9(0.37-2.22)	0.51(0.24-1.07)	**0.27(0.10-0.73)**	0.56(0.25-1.25)
**Gastrointestinal major bleeding**	0.84(0.38-1.86)	0.49(0.23-1.05)	NA	NA	NA

* The bold indicate statistically significant values.

#### Relative safety of dabigatran, apixaban, and rivaroxaban

Major bleeding was significantly lower with apixaban compared with dabigatran 150 mg BID (OR 0.19, 95% CI 0.13-0.28, p <0.0001) and rivaroxaban (OR 0.19, 95% CI 0.14-0.28, p <0.0001), but was not significantly different from dabigatran 110 mg BID (Table 3). Comparing with rivaroxaban only Dabigatran 110 mg BID, was related to significant less major bleeding (OR 0.68, 95%CI O.46-0.99, p=0.048), not dabigatran 150 mg BID. Dabigatran 110 mg BID also had lower intracranial bleeding (OR 0.27, 95%CI 0.10-0.73, p=0.01) compared with rivaroxaban. Risk of gastrointestinal major bleeding was similar with apixaban or dabigatran. 

### Meta-Regression Analysis

**Figure 6:**
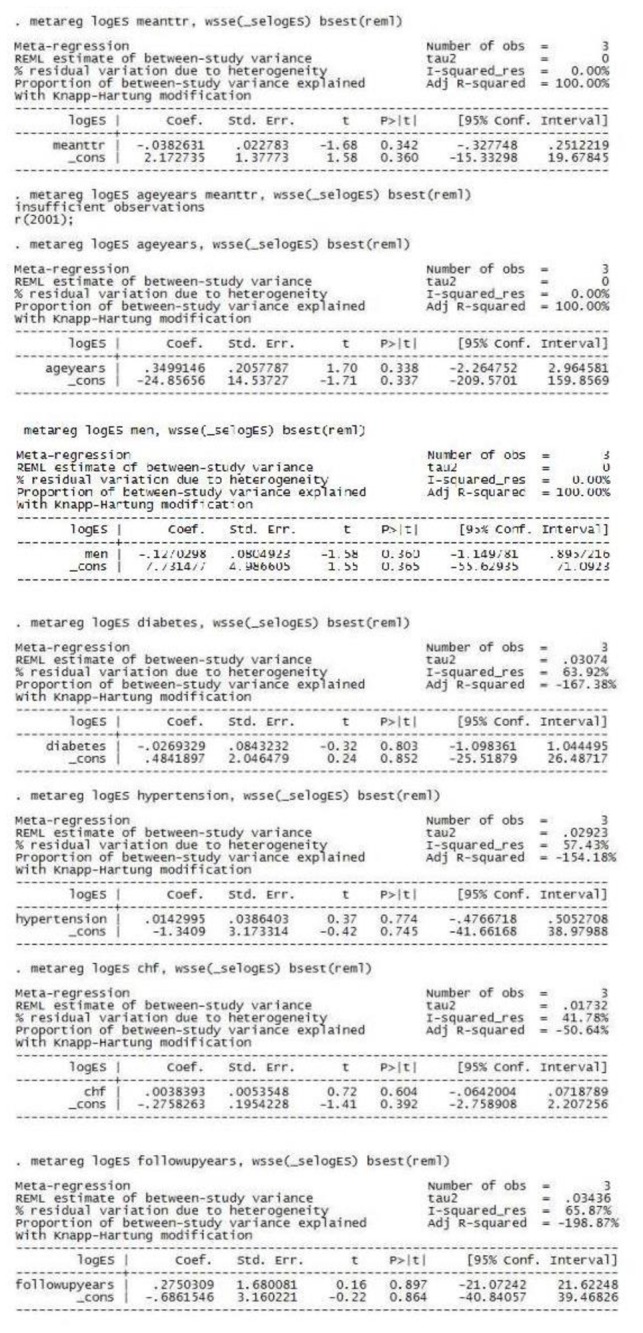
Meta-regression analysis for stroke (with following variables- mean time in therapeutic range, mean age, percentage of male population, percentage of patients with diabetes, hypertension, congestive heart failure and follow up duration).

**Figure 7:**
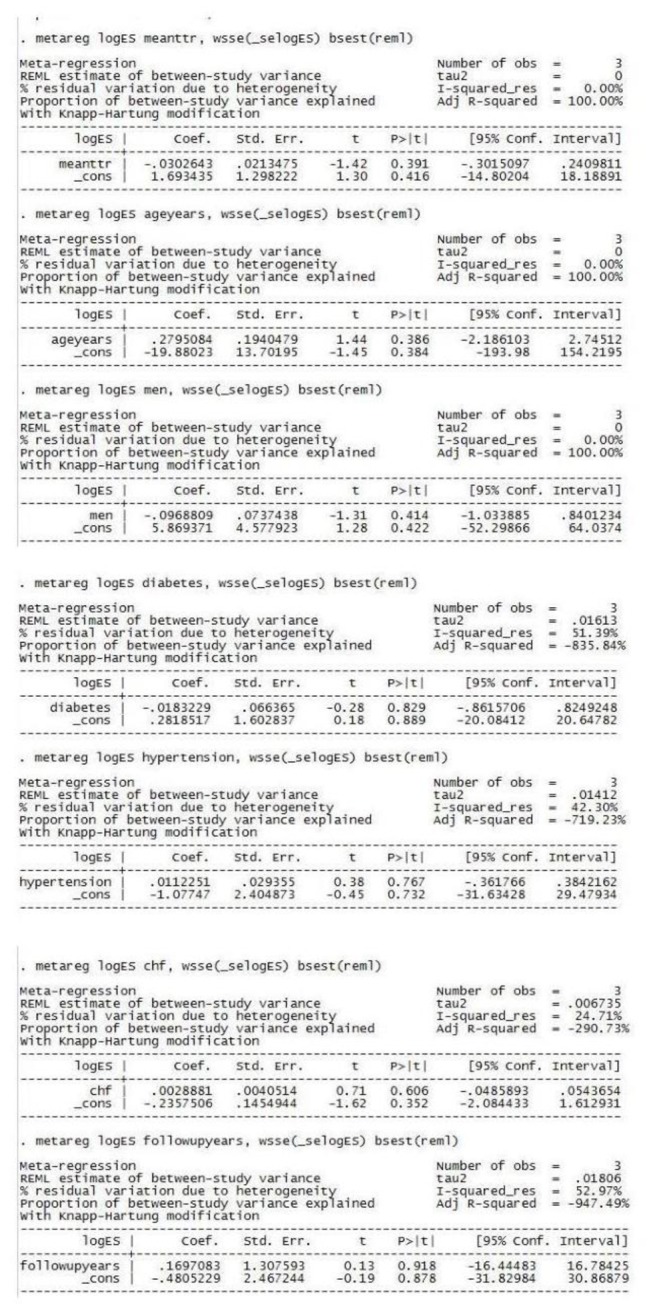
Meta-regression analysis for major bleeding (with following variables- mean time in therapeutic range, mean age, percentage of male population, percentage of patients with diabetes, hypertension, congestive heart failure and follow up duration).

To identify the impact of baseline variables and other factors on the outcomes, meta-regression analysis was performed. Mean TTR, mean age and percentage of male population, explained fully the observed heterogeneity in the risk of stroke and major bleeding, however other variables (percentage of patients with diabetes, hypertension, CHF and follow up duration) did not contribute to the heterogeneity (Figures 6 and 7). 

There was also no significant publication bias detected with examination of funnel plots or with Egger’s regression test for primary outcomes (p=0.2). Risk of bias was objectively assessed (Table 4).

**Table 4:** Risk of bias assessments for included studies.

	RE-LY	ROCKET-AF	ARISTOTLE
Random sequence generation (selection bias)	Low	Low	Low
Allocation concealment (selection bias)	Low	Low	Low
Blinding of participants and researchers (performance bias)	Unclear	Low	Low
Blinding of outcome assessment (detection bias)	Low	Low	Low
Incomplete outcome data (attrition bias)	Low	Low	Low
Selective reporting (reporting bias)	Low	Low	Low
Other bias	Low	Low	Low

## Discussion

In this systematic review and meta-analysis we found that compared to warfarin, the effectiveness of NOACs over that of warfarin, is not consistent in patients with AF and previous stroke or TIA. Our indirect comparison analysis shows that there were no significant efficacy differences between apixaban, dabigatran (150 mg BID), or rivaroxaban in patients with previous stroke. Dabigatran 110 mg BID caused less hemorrhagic stroke compared to rivaroxaban. In clinical practice, patients with AF and previous stroke or TIA are common and previous studies revealed that these patients have higher risk of death and other complications [1,19]. 

A number of recent reviews of efficacy and safety of New Oral Anticoagulants are available based on the initial RELY, ROCKET-AF and ARISTOTLE trial [16-18,20-22]. The newer oral anticoagulants were found to be more efficacious or non-inferior than warfarin for the prevention of stroke or systemic embolism and other outcomes in patients with AF (including all risk factors). Direct comparison analysis by Miller CS et al [20], showed patients randomized to NOACs had a decreased risk for all-cause stroke and systemic embolism, ischemic stroke, all-cause mortality and intracranial bleeding, however data regarding the risks for major bleeding and gastrointestinal bleeding were inconclusive. Similar results were also reported by Dogliotti A et al [21]. Adam SS et al, also reported less all-cause mortality with NOACs [22]. Meta-analysis by Kwong JS et al, including data from 13 studies showed NOACs are more effective in reducing stroke and systemic embolism without increasing the risk of major bleeding compared to conventional oral anticoagulants [23]. Dentali F et al, meta-analyzed data from 12 studies and showed, NOACs significantly reduced all-cause mortality, cardiovascular mortality, and stroke or systemic embolism. Analysis for safety outcomes showed, there was a trend toward less major bleeding and a significant reduction of intracranial hemorrhage with NOACs [24]. 

Efficacy and safety of NOACs also have been evaluated in recent network meta-analyses. A pair-wise and warfarin-controlled network meta-analyses by Biondi-Zoccai G et al [25] showed that after a weighted average of 23 months of follow up, NOACs lead to significant reductions in the risk of stroke or systemic embolism and all cause mortality in comparison to warfarin. Network meta-analysis showed that dabigatran and apixaban were similarly superior to warfarin in prevention of stroke or systemic embolism, but apixaban was related to less major bleedings compared to dabigatran. In comparison to warfarin, rivaroxaban did not reduce stroke or systemic embolism and major bleedings, rivaroxaban was also associated with excess major bleedings than apixaban. Another network meta-analysis by Harenberg J et al [26] showed, dabigatran (150 mg BID) had superior efficacy in preventing ischemic stroke or systemic embolism than dabigatran (110 mg BID) and rivaroxaban. 

We previously published the data on effectiveness of NOACs for extended treatment of venous thromboembolism [27]. NOACs were effective for the long term treatment of venous thromboembolism and might reduce the risk of all-cause mortality. Our analysis also showed that dabigatran and rivaroxaban may cause more major or clinically relevant bleeding in this population [27]. Recently we evaluated the risk of intracranial hemorrhage with NOACs in patients with atrial fibrillation. Our analysis revealed, all the three individual NOACs (dabigatran, rivaroxaban, apixaban) reduced the risk of intracranial hemorrhage, Bayesian indirect comparison analysis did not reveal a significant superiority of any specific medications [28]. 

History of previous stroke is regarded as very high risk in all Stroke Risk Stratification Schemes including CHA2DS2-VASc, NICE (2006), ACC/AHA/ESC (2006), Framingham (2003), CHADS2 (2001), ACCP (1998, 2001), SPAF (1995, 1998) and AFI (1994) [1-3,29]. In the very popular CHADS_2_ score scheme, 2 points (highest among all risk factors) is to be added for a prior stroke or TIA. Subgroup Analysis of the RE-LY Trial showed that higher CHADS_2_ scores were associated with increased risks for stroke or systemic embolism, bleeding, and death in patients with atrial fibrillation receiving newer or older oral anticoagulants [30]. Recent analyses have shown that NOACs have benefits over warfarin that is consistent across patient risk of stroke as assessed by the CHADS_2_ score. A very recent secondary analysis of the ARISTOTLE trial documented that apixaban significantly reduced stroke or systemic embolism and other outcomes, with no evidence of a differential effect by risk of stroke [31].

Interestingly, in our analysis we found that in comparison to warfarin, efficacy of NOACs in patients with AF and previous stroke or TIA is not consistent. NOACs are recommended by previous trials for use in moderate to high risk patients with previous stroke, not with very low risk patients, because all of RELY, ROCKET-AF or ARISTOTLE did not include very low risk patients (for stroke). Though the NOACs may have some benefit over warfarin in very high risk patients (patients with AF and previous stroke), but our findings did not show any benefit of NOACs in secondary prevention of ischemic stroke. Limitations aside, our meta-analysis emphasizes the need for future randomized trials focusing on the use of NOACs in AF patients with previous stroke. 

In our analysis, the risk of major bleeding was comparable between and NOACs and warfarin, and, gastrointestinal major bleeding was higher (the results to be interpreted cautiously due to wide CI) in patients with NOACs compared to warfarin. Similar findings were also reported previously [22,23]. 

Recent indirect comparison analyses, including data from initial RELY, ROCKET-AF and ARISTOTLE trials concluded that – the relative efficacy of new anticoagulants are not profoundly different [32-34]. These analyses showed dabigatran 150 mg BID was superior to rivaroxaban for the efficacy endpoints of stroke or systemic embolism. The risk of major bleeding was significantly lower with dabigatran 110 mg BID or apixaban. An analysis by Baker WL et al showed apixaban lowered the risk of major bleeding and gastrointestinal bleeding compared to dabigatran [34]. Compared to rivaroxaban, apixaban decreased the risk of major bleeding, but increased the risk of systemic emboli [34]. These analyses did not consider the separate analyses of the subgroup of patients with previous stroke. In our analysis we also found protective effects of apixaban or dabigatran 110 mg BID in the prevention of major bleeding, but there was no difference between Dabigatran 150 mg BID and rivaroxaban in efficacy endpoints in AF patients with a previous stroke or TIA. 

A recently published meta-analysis (classical network meta analysis) indirectly compared the new oral anticoagulant drugs for primary and secondary prevention of stroke in atrial fibrillation [35]. They found beneficial effects of dabigatran 110 mg BID over rivaroxaban in secondary prevention for hemorrhagic stroke, vascular death, major bleeding, and intracranial bleeding. On the other hand, the main objective of our study was to compare the efficacy of NOACs and warfarin in the secondary prevention of stroke. As mentioned earlier, our analysis revealed that in prevention of ischemic stroke, NOACs are not better than warfarin in this high risk population, but may be safer especially for the risk of ICH. This important message is missing in that article. We also found a beneficial effect of apixaban compared to rivaroxaban and dabigatran 150 mg BID for the risk of major bleeding. 

In our analysis, the only meaningful finding in support of use of NOACs in AF with previous stroke was significant reduction of hemorrhagic stroke and intracranial bleeding. But careful interpretation revealed that this benefit is limited to low dose dabigatran (110 mg BID). This drug may be a potential choice in this high risk group. But currently FDA has approved only 150 mg and 75 mg of dabigatran in the USA, and efficacy of dabigatran 75 mg BID has not been assessed in these high risk patients. There are also recent concerns regarding significant bleeding events and myocardial infarction with the use of dabigatran [36]. In this context, the risk vs. benefit of low dose dabigatran in this population should be interpreted cautiously.

## Study Limitations

Our present study has potential limitations. We included data from the subgroup analyses of initial trials, which may introduce analytic challenges [37]. Our meta-regression analysis revealed the impact of differences in patient characteristics on clinical outcomes. ROCKET-AF included a comparatively higher risk population. Patients of the warfarin group in ROCKET-AF had much less ‘mean time in therapeutic range’ than that of RE-LY and ARISTOTLE trials, which is difficult to adjust for (although we did not find a significant association with our meta-regression). Our analysis also cannot directly evaluate the effects of NOACs, for the patients who have a stroke while on-treatment with warfarin. Most of the outcomes are in the borderline zone, so that should be interpreted very cautiously. Studies with edoxaban, another NOAC was not included in the analysis-due to non-availability of the data. 

NOACs are chemically different compounds, and dabigatran which is a direct thrombin inhibitor is a different group of medication compared to factor Xa inhibitors (rivaroxaban and apixaban). So, there are concerns regarding the pooling of data for these compounds. However, this method of data pooling is an acceptable approach and several meta-analysis have been published recently with pooled analysis including these three NOACs [22–24]. Data pooling across studies increases the power of the analysis, permits a full examination of effect modification within the data and adds robustness to the results obtained [22]. 

As per the International Society for Pharmacoeconomics and Outcomes Research (ISPOR), recommendations and guidelines, in the absence of RCTs involving a direct comparison of all treatments of interest, an indirect treatment comparison provides useful information [38]. However, indirect comparison analysis has its limitations, most importantly, the failure of the assumptions of similarity and consistency may render results questionable [32,38]. 

## Conclusion

Based on available trials data, no major differences in the efficacy between warfarin and NOACs were found in AF patients with a previous stroke or TIA. But pooled analysis revealed a lower risk of ICH with use of NOACs. The relative effects of apixaban, dabigatran (both doses) and rivaroxaban are not significantly different in AF patients with a previous stroke or TIA. Randomized clinical trials focusing on this high risk group and head-to-head real-world direct comparison of the different NOACs are required to properly recognize the efficacy and safety of the different newer anticoagulants in the secondary prevention of stroke. 

## Supporting Information

Table S1 **Definitions of efficacy and safety outcomes.**
(DOCX)

Checklist S1 **PRISMA Checklist of items to include when reporting a systematic review or meta-analysis.**
(DOC)
