# Supplementary material for: New Oral Anticoagulants Are Not Superior to Warfarin in Secondary Prevention of Stroke or Transient Ischemic Attacks, but Lower the Risk of Intracranial Bleeding: Insights from a Meta-Analysis and Indirect Treatment Comparisons
Source: PLoS One. 2013 Oct 25;8(10):e77694. doi: 10.1371/journal.pone.0077694 (PMC3808395; doi:10.1371/journal.pone.0077694)
Supplement: Table S1 — Definitions of efficacy and safety outcomes. (DOCX) [file pone.0077694.s001.docx]

**Table S1: Definitions of efficacy and safety outcomes**

| Outcome Definitions | RE-LY | ROCKET AF | ARISTOTLE |
| --- | --- | --- | --- |
| Stroke | Stroke was defined as the sudden onset of a focal neurologic deficit in a location consistent with the territory of a major cerebral artery and categorized as ischemic, hemorrhagic, or unspecified. Hemorrhagic transformation of ischemic stroke was not considered to be hemorrhagic stroke. Intracranial hemorrhage consisted of hemorrhagic stroke and subdural or subarachnoid hemorrhage. | A sudden focal neurological deficit of presumed cerebrovascular etiology that persisted beyond  24 hours and was not due to another identifiable cause. An event matching this definition but lasting less than 24  12  hours was considered to be a transient ischemic attack. Brain imaging (computed tomography or magnetic resonance  imaging) was recommended for all suspected strokes, and this was performed in 82.1% of patients with strokes. | Stroke was defined as a non-traumatic focal neurologic deficit lasting ≥24 hours.  A retinal ischemic event (embolism or thrombosis) was considered a stroke. A  cerebral imaging study (computed tomographic scan or magnetic resonance  imaging) was recommended for all suspected strokes.  1. Strokes were classified as ischemic, ischemic with hemorrhagic  transformation, hemorrhagic, or of uncertain type. Hemorrhagic strokes  were sub-classified as subdural, subarachnoid, or intra-parenchymal.  2. A transient ischemic attack (TIA) was defined as a non-traumatic abrupt  onset of a focal neurologic deficit lasting <24 hours.  Stroke and TIA were further sub-classified based on whether there was imaging  evidence of a new cerebral infarction that correlated with the clinical presentation  of the subject. |
| Systemic embolism | Systemic embolism was defined as an acute vascular occlusion of an extremity or organ, documented by means of imaging, surgery, or autopsy. | Abrupt vascular insufficiency associated with clinical or radiological  evidence of arterial occlusion in the absence of another likely mechanism (e.g., atherosclerosis, instrumentation, or  trauma) | The diagnosis of systemic embolism required a clinical history consistent with an  acute loss of blood flow to a peripheral artery (or arteries) supported by evidence  of embolism from surgical specimens, autopsy, angiography, vascular imaging,  or other objective testing. |
| Major bleeding | Major bleeding was defined as a reduction in the hemoglobin level of at least 20 g per liter, transfusion of at least 2 units of blood, or symptomatic bleeding in a critical area or organ. | Clinically overt bleeding associated with any of the following: fatal  outcome, involvement of a critical anatomic site (intracranial, spinal, ocular, pericardial, articular, retroperitoneal, or  intramuscular with compartment syndrome), fall in hemoglobin concentration >2 g/dL, transfusion of >2 units of  whole blood or packed red blood cells, or permanent disability | Major bleeding was defined as acute or sub-acute clinically overt bleeding  accompanied by ≥1 of the following:  1. a decrease in hemoglobin level of ≥2 g/dL  2. a transfusion of ≥2 U of packed red blood cells  3. bleeding that was fatal or occurred in the following critical sites: intracranial,  intra-spinal, intra-ocular, pericardial, intra-articular, intra-muscular  with compartment syndrome, retroperitoneal. |
